# Characterising the prognostic potential of HLA-DR during colorectal cancer development

**DOI:** 10.1007/s00262-020-02571-2

**Published:** 2020-04-18

**Authors:** Margaret R. Dunne, James J. Phelan, Adriana J. Michielsen, Aoife A. Maguire, Cara Dunne, Petra Martin, Sinead Noonan, Miriam Tosetto, Robert Geraghty, David Fennelly, Kieran Sheahan, Elizabeth J. Ryan, Jacintha O’Sullivan

**Affiliations:** 1 Department of Surgery, Trinity Translational Medicine Institute, Trinity Centre for Health Sciences, St. James’s Hospital, Dublin 8, Ireland; 2 Department of Histopathology, Trinity College, St. James’s Hospital, Dublin 8, Ireland; 3 Centre for Colorectal Disease, Education and Research Centre, St. Vincent’s University Hospital, Elm Park, Dublin 4, Ireland; 4 Health Research Institute, Department of Biological Sciences, University of Limerick, Limerick, Ireland

**Keywords:** HLA-DR, Colorectal cancer, Adenoma, Cancer immunology, Prognostic biomarkers, Cancer recurrence

## Abstract

HLA-DR, an MHC class II molecule that mediates antigen presentation, is a favourable prognostic indicator in colorectal cancer (CRC). However, the dynamics and location of HLA-DR expression during CRC development are unclear. We aimed to define HLA-DR expression by immunohistochemistry in colorectal epithelium and stromal tissue at different stages of cancer development, assessing non-neoplastic colorectal adenocarcinoma–adjacent tissue, adenomas and carcinoma tissues, and to associate HLA-DR levels with clinical outcomes. Patients with higher than median HLA-DR expression survived at least twice as long as patients with lower expression. This association was significant for HLA-DR staining in the colorectal carcinoma epithelium (*n* = 152, *p* = 0.011, HR 1.9, 95% CI 1.15–3.15) and adjacent non-neoplastic epithelium (*n* = 152, *p* < 0.001, HR 2.7, 95% CI 1.59–4.66), but not stroma. In stage II cases, however, the prognostic value of HLA-DR expression was significant only in adjacent non-neoplastic tissues, for both epithelium (*n* = 63, *p* = 0.015, HR 3.6, 95% CI 1.279–10.25) and stroma (*n* = 63, *p* = 0.018, HR 5.07, 95% CI 1.32–19.49). HLA-DR was lower in carcinoma tissue compared to matched adenomas (*n* = 35), in epithelium (*p* < 0.01) and stroma (*p* < 0.001). HLA-DR was further reduced in late-stage carcinoma (*n* = 101) compared to early stage (*n* = 105), in epithelium (*p* < 0.001) and stroma (*p* < 0.01). HLA-DR expression was lower (*p* < 0.05) in the adjacent non-neoplastic epithelium of patients with cancer recurrence. We demonstrate a progressive loss of HLA-DR in epithelial and stromal tissue compartments during CRC development and show prognostic ability in carcinoma–adjacent non-neoplastic tissues, highlighting the importance of this molecule in the anti-cancer immune response. These findings may have wider implications for immunotherapeutic interventions.

**Electronic supplementary material:**

The online version of this article (10.1007/s00262-020-02571-2) contains supplementary material, which is available to authorized users.

## Introduction

Colorectal cancer (CRC) is the third most common type of cancer worldwide and the third leading cause of cancer mortality [1]. Despite recent advances in treatment and pathological staging, many CRC patients still experience cancer recurrence. Most notably, 25% of stage I/II cancers detected at the node-negative, metastasis-negative stage experience cancer recurrence [2]. Therefore, there is an urgent need to improve current prognostic and surveillance strategies for CRC patients and to better understand the factors underlying disease progression and recurrence. In recent years, certain immune markers, such as CD3, CD8 and CD45RO, have been shown to play a favourable prognostic role in CRC, highlighting the requirement for effective immune responses for cancer elimination [3–5]. The prognostic potential of other immune markers upstream of the adaptive immune response is also of great interest, such as the antigen presentation molecule HLA-DR.

HLA-DR is a major histocompatibility complex (MHC) class II antigen presentation molecule, critical for the activation of lymphocytes and the orchestration of adaptive immune responses. HLA-DR is normally expressed on antigen-presenting cells including monocytes, macrophages, dendritic cells and B cells, but expression can be induced on epithelial cells and tumour cells in response to inflammatory conditions [6–8]. MHC class II antigen presentation molecules are responsible for presentation of exogenous antigens to CD4^+^ helper T cells. Unsurprisingly, HLA-DR expression in tumours has been positively associated with enhanced lymphocytic infiltration [9–12]. HLA-DR has been used as a surrogate marker of immune competence in various clinical studies, and its loss in circulation has been linked with susceptibility to post-surgical infection [13] and sepsis [14]. The mechanism that drives this reduction in monocyte HLA-DR is not well understood, but this phenomenon has been described in response to even minor surgeries. It has been suggested that restoration of HLA-DR expression, as opposed to the initial loss itself, may be a more useful predictor of post-operative complications [15].

In the neoplastic setting, HLA molecules have demonstrated a largely favourable prognostic role in gastrointestinal cancers, such as oesophageal adenocarcinoma [16], cancer of the larynx [17], gastric cancer [18] and CRC [19, 20]. It has been proposed that the improved patient survival observed in HLA-DR expressing tumours reflects an active anti-tumour immune response, as evidenced by the correlation between HLA expression and intra-tumoral lymphocytic infiltration [11, 19]. Despite previous descriptions of HLA molecule expression in tumours and its clear clinical implications, few studies have evaluated HLA-DR expression specifically during cancer development, and fewer still have defined expression within different tissue compartments, i.e. epithelium versus stromal regions, instead giving generalised staining scores.

This study aimed to evaluate changes in HLA-DR expression during development of CRC from colorectal adenomas and adenocarcinomas at early and late stages and to define expression within both epithelial and stromal tissue compartments, and in both carcinoma and carcinoma–adjacent tissues. HLA-DR expression was evaluated by immunohistochemistry using archival formalin-fixed, paraffin-embedded colorectal tissue resections. We aimed to investigate HLA-DR loss in CRC cancer progression and to determine whether this is a universal phenomenon, common to all carcinomas, or whether HLA-DR loss only occurred in certain patients. We were also interested in determining which colorectal tissue compartments expressed the highest levels of HLA-DR and which experienced most HLA-DR loss. Finally, we were interested in determining whether HLA-DR expression still showed a prognostic ability even in early cancer stages, or in non-neoplastic carcinoma–adjacent colorectal tissues.

## Materials and methods

### Patient cohorts and specimens

For this study, formalin-fixed paraffin-embedded colorectal tissue specimens (all histologies) were collected from patients undergoing surgical resection or polypectomy at St Vincent’s University Hospital, Dublin. Tissue regions of interest were defined by a pathologist (AAM). Carcinoma–adjacent non-neoplastic colorectal tissue specimens were also collected from areas at least 10 cm away from the primary carcinoma. Tumour specimens were sampled from the primary tumour site. This study was performed in adherence to the Declaration of Helsinki ethical principles for medical research involving human subjects. Written informed consent was obtained in accordance with local institutional ethical guidelines. Full ethical approval to conduct this study was grant by the St. Vincent's University Hospital ethics committee.

#### Adenomas

Adenomas (isolated, synchronous or contiguous) were collected from a cohort of *n* = 79 patients (*n* = 25 male, mean age 70.2 [range 48–86]) undergoing treatment at St Vincent’s University Hospital, Dublin, between the years 1994–2006. Percentage HLA-DR-positive staining scores were available for matched carcinoma specimens for *n* = 33 synchronous and *n* = 35 contiguous adenomas. Synchronous adenomas were defined as adenomas separate from the main carcinoma identified in the resection specimens, while contiguous adenomas were defined as residual adenoma contiguous with the primary carcinoma.

#### CRC cohort—all stages

Carcinoma and adjacent tissues were collected from a cohort of *n* = 152 patients diagnosed with CRC who underwent surgical resection at St Vincent’s Hospital, Dublin. This cohort was sub-divided by stage using the American Joint Committee on Cancer TNM staging system for subsequent studies, details as follows.

#### CRC stage II cohort

As detailed in Table 1, a cohort of *n* = 77 CRC carcinomas (*n* = 34 female; *n* = 43 male) with a diagnosis of stage II was compiled from retrospective archival tissue collected between 1991 and 2001 from patients treated at St Vincent’s University Hospital, Dublin. Median patient age at diagnosis was 73.5 (range 48–91). Patients were followed from the date of diagnosis until death or median follow-up time of 6.1 years. Incidence of all deaths and disease recurrence was recorded during the follow-up period. Overall survival was defined as time from diagnosis until death, from any cause.

**Table 1:** Stage II CRC patient cohort

Parameter	Category	Frequency	Total
Gender	Female	34	77
	Male	43	
Lymphovascular invasion	Positive	15 (1 mural)	77
	Negative	46	
	Not reported	16	
Pathological T stage	1	0	77
	2	0	
	3	58	
	4	8	
	Not reported	11	
Differentiation	Well	4	77
	Moderate	54	
	Poor	7	
	Undocumented	12	
HLA-DR expression (mean % positivity)	Normal epithelium	42.3%	63*
	Carcinoma epithelium	44%	63*
	Normal stroma	61.1%	77
	Carcinoma stroma	49.3%	77

*Denotes missing values due to tissue degradation or missing tissue microarray cores

#### CRC late-stage cohort

As detailed in Table 2, a cohort of *n* = 75 CRC carcinoma patients (*n* = 40 male; *n* = 35 female) with a diagnosis of stage III or IV (Duke’s C and D) was compiled from retrospective archival tissue collected between from patients treated at St Vincent’s University Hospital, Dublin. Median patient age at diagnosis was 65.6 years (range 25.4–83.8 years). Patients were followed from the date of diagnosis until death or median follow-up time of 2.04 years. Overall survival was defined as time from diagnosis until death, from any cause.

**Table 2:** Late-stage patient cohort

Parameter	Category	Frequency	Total
Gender	Male	40	
	Female	35	75
Nodal stage	0	22	
	1a	6	75
	1b	15	
	2a	16	
	2b	16	
T stage	1	1	
	2	1	75
	3	33	
	4	40	
Differentiation	Well	5	
	Moderate	54	75
	Poor	15	
	Undocumented	1	
HLA-DR expression (mean % positivity)	Normal epithelium	4%	71*
	Carcinoma epithelium	9.3%	70*
	Normal stroma	60.7%	72*
	Carcinoma stroma	36%	72*

*Denotes missing values due to tissue degradation or missing tissue microarray cores

### HLA-DR immunohistochemistry analysis using tissue microarrays

Haematoxylin and eosin (H&E)-stained slides from formalin-fixed, paraffin-embedded biopsies were used to define areas of adenoma, carcinoma or adjacent non-neoplastic tissues by a pathologist (AAM). The areas of interest on the diagnostic biopsy blocks were marked by a pathologist (AAM), four 0.6 mm cores were taken from the tissue blocks, transferred to a recipient block using a Tissue Microarrayer (Beecher Instruments), and tissue microarrays (TMAs) were constructed and pathology of the tissue re-evaluated prior to antibody staining. Four cores were taken from each individual tissue type (tumour, polyp, etc.), and a 4-μM section from each was included on the TMA slide in order to reduce sampling errors, and to check that staining patterns were consistent within each tissue area. Each section was scored individually; then these four scores were averaged to give an overall score. A consistent staining pattern was observed for each tissue area sampled, with the majority of scores being either identical, or falling within 1 scoring increment. Different tissue types were run together on the same TMA slide, in order to minimise technical artefacts. Four-micrometre sections were cut and mounted onto Super Frost Plus adhesive slides (Menzel-Glaser, Germany). Immunohistochemistry was performed utilising a Vectastain Elite ABC HRP kit (Vector Laboratories, USA) or DAKO ChemMate Envision kit (Dako, Denmark), as per manufacturer’s instructions. Tissue staining was compared between TMA slides to ensure similar staining patterns, and intensities were observed between kits. All tissue microarray sections for each patient cohort were processed and stained on the same day at room temperature.

Staining was performed as previously described [16], using the monoclonal mouse anti-HLA-DR IgG (clone TAL 1B5, Abcam, Cambridge, UK) primary antibody. To control for potential cross reactivity, whole full-face tissue sections were incubated with PBS in the absence of primary antibody and/or secondary antibody. Slides were incubated with primary antibody for 1 h, biotinylated secondary antibody for 30 min, avidin–biotin complex for 30 min and diaminobenzidine (DAB) substrate for 2–15 min. DAB was rinsed off slides upon colour development and haematoxylin added for 30 s. Slides were dehydrated and mounted using DPX. Images were taken using a ScanScope GL digital slide scanner using Aperio ImageScope software (Aperio Technologies, USA), and immunoreactivity was assessed digitally under 40X magnification in a semi-quantitative manner for visible HLA-DR expression by observers (MRD, JJP, AJM) who were blinded to the pathology and clinical outcome of all patients in the study. Epithelium was defined by columnar shape, and all other non-epithelial tissue was defined as stroma; both epithelial and stromal cells were evaluated for both percentage positivity of cytoplasmic staining. HLA-DR positivity was evaluated as 0%, 10%, 25%, 50%, 75%, 90% or 100% of total visible tissue compartment. The scoring was undertaken by consensus evaluation (MRD, JJP, AJM), and scores from all observers were then averaged to create a final consensus score. Cohorts were divided into HLA-DR high and low populations by median positivity scores. We adhered to the REMARK guidelines [21] for reporting prognostic markers in order to allow reproducibility of our findings.

### Statistical analyses

Statistical analyses were performed and graphed using Prism GraphPad (Version 5) and IBM SPSS Statistics (Version 24). For survival analysis, the median percentage score of the cohort was chosen as a cut-off value in order to segregate HLA-DR high (> median) and low (0-median, inclusive) staining levels. Survival was analysed utilising Kaplan–Meier survival analysis (log-rank (Mantel–Cox)). Paired tests were used for matched tissues from the same donors, as appropriate. Differences of *p* < 0.05 (*), *p* < 0.01 (**) and *p* < 0.001 (***) were considered statistically significant.

## Results

### HLA-DR expression is associated with a longer survival time when measured in carcinoma and carcinoma–adjacent epithelium, but not in stroma

**Fig. 1:**
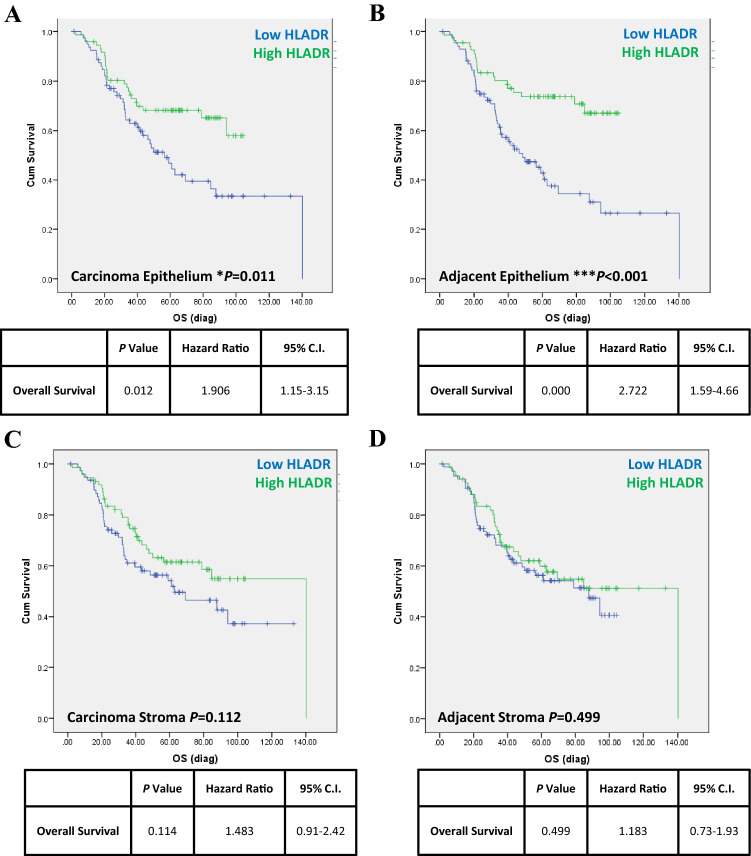
HLA-DR expression is associated with longer overall survival when measured in carcinoma and carcinoma–adjacent epithelium, but not in stroma. Data from *n* = 152 CRC patients at all stages were combined and analysed utilising Kaplan–Meier survival analysis (log rank (Mantel–Cox)). HLA-DR expression profiles were divided into high and low categories, using median expression as a cut-off. High HLA-DR expression in the epithelial compartments of **a** carcinoma (HR 1.9, *p* = 0.011, high *n* = 72, low *n* = 80) and **b** matched carcinoma–adjacent normal tissue (HR 2.7, *p* < 0.001, high *n* = 67, low *n* = 85) was associated with improved overall survival. No statistically significant associations were detected between survival and HLA-DR expression profiles in stromal tissues from either **c** carcinoma stroma (*p* = 0.112, high *n* = 73, low *n* = 79) or **d** matched carcinoma–adjacent normal tissue stroma (*p* = 0.499, high *n* = 67, low *n* = 85). Cox regression multivariate analysis demonstrated that, in addition to low HLA-DR expression, T stage 4 (*p* = 0.02), moderate differentiation stage (*p* = 0.001) and poor differentiation stage (*p* < 0.000) were all predictors of poor survival, associated with twofold, 2.8-fold and sevenfold increases in disease-associated death, respectively. *p < 0.001, ***p < 0.0001

We first investigated whether HLA-DR expression in epithelial and stromal tissue compartments was linked with patient survival, in carcinoma and carcinoma–adjacent colorectal tissues at all tumour stages. HLA-DR-positive expression was assessed in a cohort of *n* = 152 CRC patients at all carcinoma stages (demographics as per Tables 1 and 2). Patients were assigned to HLA-DR high or low groups, using median expression level as a cut-off, and associations were tested by log rank (Mantel–Cox) tests and estimated using Kaplan–Meier survival curves. As shown in Fig. 1, we observed a longer survival time in patients expressing high levels of HLA-DR in the tissue epithelium, both in the carcinoma epithelium (Fig. 1a) (*p* = 0.012, HR 1.91, 95% CI 1.2–3.2, *n* = 72) and in carcinoma–adjacent epithelia (Fig. 1b) (*p* < 0.001, HR 2.72, 95% CI 1.6–4.7, *n* = 67), compared to those with lower than median expression (*n* = 80 and *n* = 85, respectively). No significant associations were observed when HLA-DR expression in the carcinoma stroma (Fig. 1c) (*p* = 0.11) or carcinoma–adjacent stroma (Fig. 1d) (*p* = 0.5) was analysed, however. Cox regression multivariate analyses also demonstrated prognostic ability of other parameters, with T stage 4 (*p* = 0.02, HR 2), moderate differentiation (*p* = 0.001, HR 2.8) and poor differentiation stage (*p* < 0.000, HR 7), and carcinoma size (*p* = 0.03, HR 1.15), all predicting increased risk of disease-associated death (Table 3).

**Table 3:** Results of cox regression (multivariate analysis) on late-stage cohort

Parameter	HR	CI	*p* value
Differentiation moderate	2.3	1.2–4.5	**0.012**
Differentiation poor	4.5	2–10.4	**0.0001**
Carcinoma size (cm)	1.14	1.01–1.3	**0.031**
T stage 4	2	1.13–3.5	**0.017**
Node stage 1 (0–2)	–	–	0.32
Node stage 2 (0–2)	–	–	0.073
Node stage 1b	–	–	0.437
Node stage 2a	–	–	0.077
Node stage 2b	–	–	0.584
HLA (normal epithelium)	–	–	0.664
HLA (normal stroma)	–	–	0.277
HLA (carcinoma epithelium)	–	–	0.99
HLA (carcinoma stroma)	–	–	0.89
No. of nodes	–	–	0.584

Bolded *p* values < 0.05 were deemed statistically significant

–Indicates no HR or CI reported during statistical analysis when *p* > 0.05

*HR* hazard ratio, *CI* confidence interval

### HLA-DR expression diminishes during CRC development

**Fig. 2:**
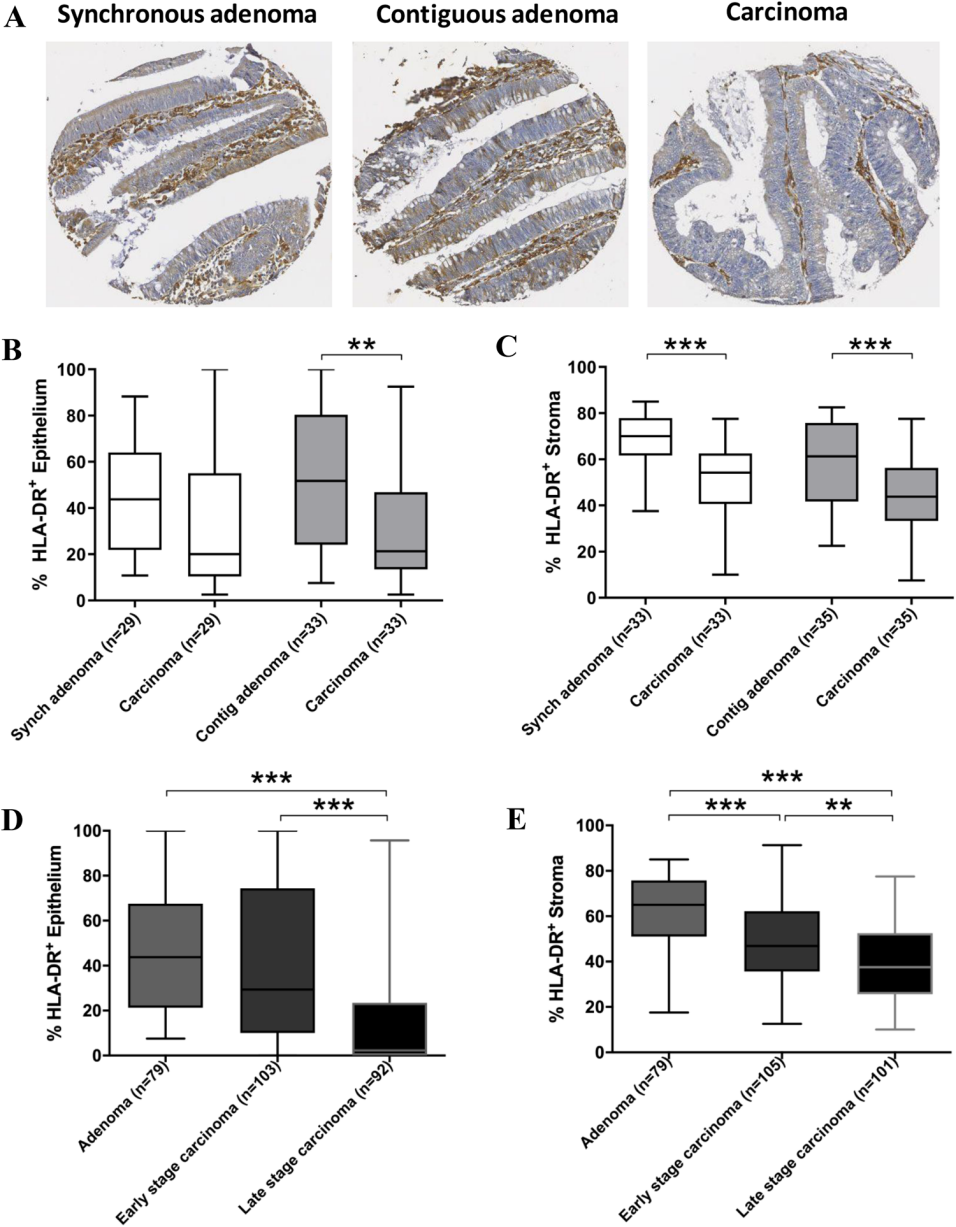
HLA-DR expression diminishes as adenomas progress to carcinomas and advance in stage. HLA-DR expression was visualised by immunohistochemistry, as shown for a single representative donor (**a**) and percentage positive expression was scored for colorectal tissue epithelium and stroma in a cohort of synchronous (*n* = 29–33) or contiguous (*n* = 33–35) adenomas and matched carcinomas (**b**, **c**) and adenomas (*n* = 79), early-stage (stage I/II, *n* = 103–105) and late-stage carcinomas (stage III/IV, *n* = 92–101) (**d**, **e**). Data were analysed using paired t test or one-way ANOVA as appropriate (Kruskal–Wallis test followed by Dunn’s multiple-comparison test). **p < 0.01, ***p < 0.001

The percentage of HLA-DR-positive expression was evaluated in CRC carcinomas and synchronous (*n* = 33) or contiguous (*n* = 35) adenomas from matched CRC patients (Fig. 2) to assess changes in HLA-DR expression at different stages in carcinoma development. HLA-DR expression was lower in carcinoma epithelium compared to the epithelium of contiguous matched adenomas (*p* < 0.01), but no difference in expression was observed when carcinomas were compared with synchronous adenomas (Fig. 2b). HLA-DR expression was lower in the CRC carcinoma stroma compared to that of both synchronous (*p* < 0.001) and contiguous adenomas stroma (*p* < 0.001) (Fig. 2c).

We next aimed to assess whether HLA-DR expression was altered across different stages of cancer development, from pre-neoplastic, to early-stage to late-stage carcinomas. HLA-DR was therefore assessed in epithelial and tumour areas of a cohort of mixed adenomas (isolated, synchronous and contiguous adenomas, *n* = 79), early-stage CRC carcinomas (*n* = 105, stage I or II) and late-stage CRC carcinomas (*n* = 101, stage III or IV) (Fig. 2d, e). In the epithelial compartment (Fig. 2d), HLA-DR expression was lower in late-stage carcinomas compared to early stage (*p* < 0.001) and adenomas (*p* < 0.001), but no significant difference was observed between adenomas and early-stage carcinomas. In the stromal compartment (Fig. 2e), HLA-DR expression was progressively reduced when late-stage carcinomas were compared with early-stage carcinomas (*p* < 0.01) or adenomas (*p* < 0.001). For both compartments, HLA-DR expression was diminished in late-stage carcinomas when compared to adenomas (*p* < 0.001).

### HLA-DR expression is lower in late-stage CRC stroma compared to carcinoma–adjacent stroma

**Fig. 3:**
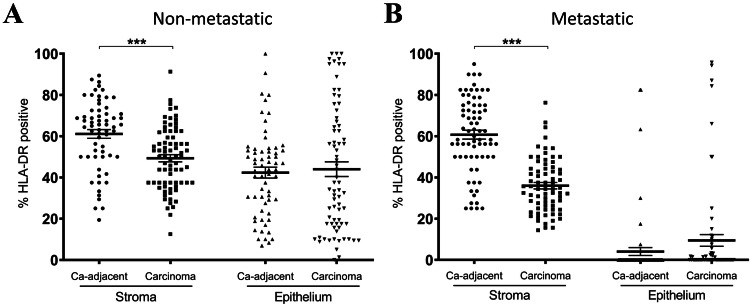
HLA-DR expression is lower in CRC carcinomas compared to carcinoma–adjacent tissue, but only in the stromal compartment. HLA-DR expression was higher in the stroma of carcinoma–adjacent tissues compared to matched carcinomas, for both early-stage **a** (*p* < 0.001, *n* = 76) and late-stage **b** (*p* < 0.001, *n* = 72) CRC cases, whereas no statistically significant difference was detected within the epithelium, regardless of stage. Wilcoxon matched paired t tests; bars denote mean ± SEM. ***p < 0.001

Having observed a significant reduction in HLA-DR expression in carcinoma tissues in later-stage carcinomas compared to early-stage, we were interested in investigating whether carcinoma–adjacent tissues were similarly affected. HLA-DR expression was assessed across the epithelial and stromal compartments of *n* = 76 early-stage (stage II) (Fig. 3a) and *n* = 72 late-stage (stage III and IV) (Fig. 3b) CRC carcinomas and adjacent non-carcinoma tissues. HLA-DR expression was lower in CRC carcinoma stroma compared to non-carcinoma stroma (*p* < 0.001), regardless of cancer stage. However, no significant difference was noted between HLA-DR expression in the epithelial compartments of carcinoma versus carcinoma–adjacent tissue, at neither early nor late stages. The profound stage-dependent loss of epithelial HLA-DR expression observed in Fig. 2 was also recapitulated in this cohort, with adjacent non-carcinoma tissue epithelium also exhibiting significantly lower HLA-DR expression in late-stage patients in comparison with early stage.

### HLA-DR clinical correlations in stage II CRC

**Fig. 4:**
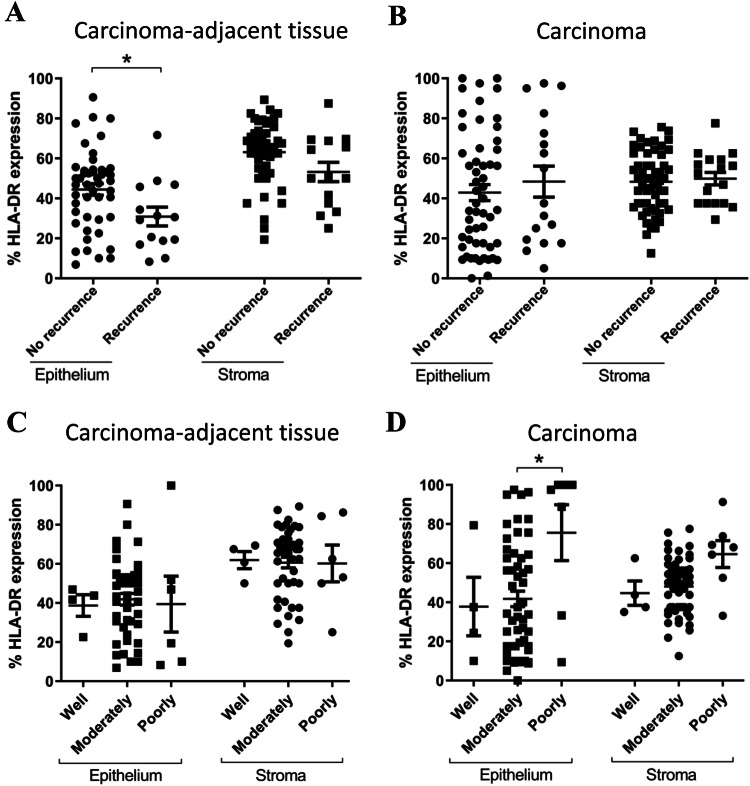
HLA-DR expression is lower in carcinoma–adjacent epithelial tissue in stage II CRC patients who experience carcinoma recurrence and highest in poorly differentiated carcinomas. HLA-DR expression was lower (*p* < 0.05) in the epithelium of stage II CRC patients experiencing a carcinoma recurrence (*n* = 14) compared to those with no recurrence (*n* = 47). This difference was only observed in the epithelium of carcinoma–adjacent tissue (**a**), and not in carcinoma epithelium or stroma (**b**). HLA-DR expression was highest in CRC carcinomas with the poorest differentiation scores, but no change was observed in carcinoma–adjacent tissues. One-way ANOVA (Kruskal–Wallis test with Dunn’s multiple comparisons) *p < 0.05

Given the significant expression differences observed between stage II carcinomas and adjacent tissues, we next investigated whether HLA-DR was linked with a set of clinical outcomes in early-stage carcinomas. HLA-DR percentage positivity was assessed in a cohort of *n* = 77 stage II CRC carcinomas (Table 1), and associations were tested with carcinoma recurrence (data available for *n* = 61 patients), lymphovascular invasion status (*n* = 61), number of carcinoma buds (*n* = 30) and carcinoma differentiation stage (*n* = 66). HLA-DR expression was compared within the stage II CRC cohort between patients who experienced carcinoma recurrence (*n* = 14) versus those who did not (*n* = 47) (Fig. 4). HLA-DR was higher in the epithelium of patients with no recurrence compared to those with recurrence (*p* < 0.05); however, this was only observed in carcinoma–adjacent colorectal tissue (Fig. 4a), and not in carcinoma tissues (Fig. 4b). No significant differences were noted within the respective stromal compartments. No significant associations were noted between HLA-DR expression and lymphovascular invasion status or number of carcinoma buds (Supplementary Figs. 1 and 2).

### HLA-DR expression in stage II carcinoma–adjacent tissue is positively associated with survival time

**Fig. 5:**
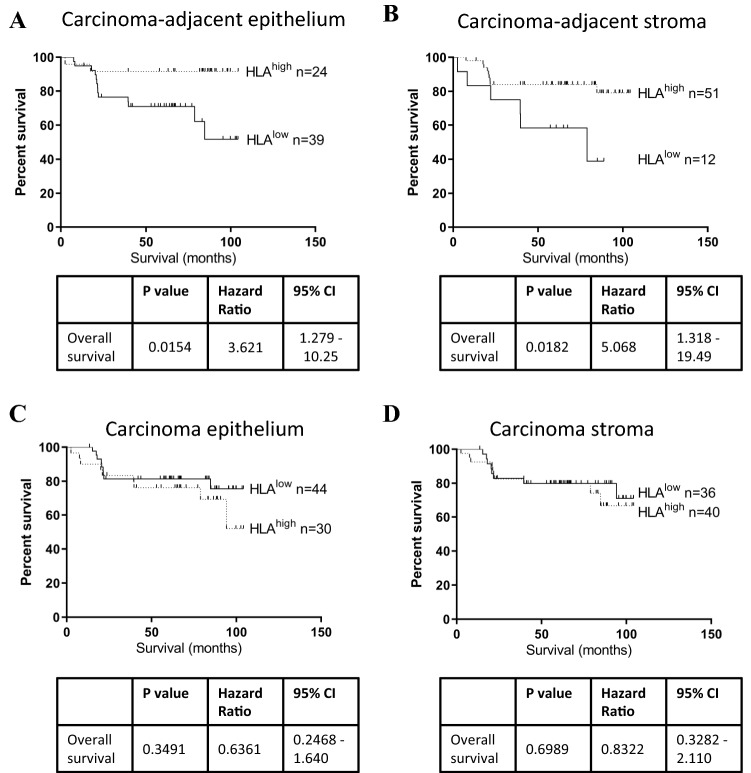
HLA-DR expression is associated with longer overall survival when measured in carcinoma–adjacent epithelium and stroma, but not in carcinoma compartments. A cohort of *n* = 77 stage II CRC patient specimens were segregated into HLA-DR high or low populations based on median expression level, and differences in survival time were assessed by log rank (Mantel–Cox) test, in tissue epithelium and stroma. The HLA-DR^high^ cohort showed a longer survival time compared to HLA-DR^low^ populations, in carcinoma–adjacent normal colorectal epithelium (**a**) and stroma (**b**), but no difference was observed within the carcinoma epithelium (**c**) or stroma (**d**)

HLA-DR expression by carcinoma or carcinoma–adjacent tissue was assessed with respect to survival time in a cohort of *n* = 77 early-stage (stage II) CRC patients (Table 1). Patients were assigned to HLA-DR high or low groups, using median expression as a cut-off. Survival times were assessed by log rank (Mantel–Cox) tests and estimated using Kaplan–Meier survival curves, as shown in Fig. 5. We observed that individuals with high HLA-DR expression in carcinoma–adjacent tissues survived longer than those with low expression, both in the tissue epithelium (*p* = 0.015, HR 3.62, 95% CI 1.3–10.3) and stroma (*p* = 0.018, HR 5.07, 95% CI 1.3–19.5), (Fig. 5a, b). No significant difference was noted in the CRC carcinoma epithelium or stroma, however (Fig. 5c, d). Therefore, HLA-DR only displayed prognostic ability in early-stage CRC when measured in carcinoma–adjacent tissues, but not in early-stage carcinomas themselves (Table 3).

## Discussion

Previous studies have demonstrated a significant association between MHC class II expression in CRC tumours and overall survival time [11, 19, 20, 22, 23], lower recurrence [24, 25], lower tumour stage [26], lymphocytic infiltration and microsatellite instability [23]. However, there is a lack of consistency in the literature regarding these findings in CRC, with several of these studies showing contradictory results, such as the level of HLA-DR in CRC tumours which has been reported as being expressed by between 23 and 60.1% of CRC tumours, depending on the study. A possible explanation for these conflicts may lie in the differences in tissue scoring methodology and reporting approaches, as well as use of MHC class II antibodies with different specificities. For example, Matsushita and colleagues analysed HLA-DR using the HU-20 antibody in 51 snap-frozen CRC tumours and adjacent tissues and defined HLA-DR positivity as cases where > 10% cancer cells were strongly stained [20]. Sconnochia*et al.,* however, used a LGII-612.14 antibody specific for all MHC class II molecules (HLA-DR, HLA-DP and HLA-DQ) to stain formalin-fixed paraffin-embedded tissue, and defined positivity as > 15 positive cells observed in a field count of 100 cells [19]. In this study, we focused on assessing expression of the MHC class II molecule HLA-DR (clone TAL 1B5) in a tissue microarray constructed from multiple tumour samples from an archival cohort of formalin-fixed paraffin-embedded colorectal carcinoma cores, and extended analyses to include patient-matched pre-malignant adenomas and non-neoplastic carcinoma–adjacent tissues, evaluating expression in both epithelial and stromal regions. We reported overall percentage positivity for the epithelium and stromal compartments, taking an average reading of three entire 0.6 mm fields into account.

In agreement with previous studies in CRC [11, 19, 20, 22, 23], we observed a significant association between higher HLA-DR expression in CRC carcinomas and overall survival, particularly in the epithelial tissue compartment. We also observed that higher HLA-DR expression in carcinoma–adjacent epithelium was positively associated with overall survival time, and this finding was particularly evident when stage II carcinomas were assessed. Carcinoma–adjacent epithelium also showed higher HLA-DR expression in patients who had not experienced cancer recurrence, signifying an important prognostic capability of non-carcinoma tissues. Other recent studies have also demonstrated the prognostic importance of extra-tumoral tissues, in different cancer settings. Prasanna and colleagues observed significant prognostic ability of ten radiographic features (so-called radiomics) in the peri-tumoral areas of glioblastoma [27]. Genetic and molecular analyses of carcinoma–adjacent histologically normal breast tissue revealed RNA signatures associated with significantly worse 10-year survival in estrogen receptor-positive breast cancer [28]. Therefore, it is plausible that immune markers outside CRC tissues demonstrate prognostic potential. Why this is particularly apparent in early-stage carcinomas is currently unclear but may reflect early immune cell trafficking into carcinomas. Further work is required to fully assess the impact of HLA-DR expression patterns in relation to clinical outcomes. We sampled expression in polyp and carcinoma cores and found relatively homogenous expression between cores; however, expression may be different at the invasive margins, and perhaps even more informative. Foukas and colleagues report differences in HLA-DR expression in lung cancer at different distances to the tumour and posit that this decrease in expression may be driven by soluble tumour factors and contribute to immunosuppression [29].

Horie and colleagues report greater HLA-DR expression in less well differentiated CRC carcinomas, although only ten carcinomas were examined [30]. We observed that HLA-DR expression was highest in poorly differentiated stage II carcinomas; however, no difference was observed in the late-stage CRC cohort.

We observed a progressive loss of HLA-DR as CRC developed from adenomas, which was also reported in other studies [24, 31]. This contrasts the findings of Sconnochia and colleagues (2014) who describe a greater percentage of MHC class II positive cells in CRC tissue, compared to adenomas or normal adjacent tissue [19]. This disparity may be explained by the different respective specificities of antibodies used and scoring methods employed, as outlined previously. In addition, our observed reduction of HLA-DR upon CRC progression from adenoma to carcinoma reflects the loss of the HLA-DR molecule itself but may also indicate a loss of the factors driving HLA upregulation or cell surface maintenance. Gene alterations occurring during cancer development can negatively regulate HLA expression [32, 33], for example damage to the class II transactivator (CIITA) gene, or large chromosomal deletions, as observed in diffuse large B cell lymphomas originating from immune-privileged sites [34]. Many inflammatory factors drive HLA-DR upregulation, such as cytokines, hormones and cell surface chaperones, which regulate HLA-DR expression on the cell surface [32]. Future mechanistic studies should identify and examine whether specific inflammatory mediators can drive and maintain HLA-DR expression in CRC tissue (at least in the absence of genetic changes), which could direct the development of improved targeted therapies [33].

Immunosurveillance protects the body against the development of cancer, as well as pathogenic threat, and immunodeficiency is associated with increased carcinoma incidence [35]. The link between HLA-DR expression in many gastrointestinal carcinomas and improved survival is thought to reflect effective anti-carcinoma immune involvement, i.e. activation of a robust adaptive T cell response. Other immune factors linked with favourable clinical outcomes and clinical response to immunotherapy include T helper (T_H_)1-associated genes, CD8 T cells, γδ T cells and memory T cells [3, 4, 36, 37]. Since HLA-DR mediated antigen presentation is critical for priming CD4^+^ T cell effector functions, HLA-DR can also be considered to be an integral, upstream part of this anti-carcinoma, T_H_1 axis, suggesting that future studies may benefit from inclusion of HLA-DR into prognostic scoring panels. HLA-DR can also be expressed by lymphocytes as an activation marker, and elevated expression has also been shown to predict patient response to neo-adjuvant treatment in the breast cancer setting, further demonstrating the usefulness of this molecule [38].

Whether HLA-DR expression by tumour cells results in effective antigen presentation to T cells is unclear, but studies have shown that epithelial cells expressing HLA-DR are capable of antigen presentation [7] and cross-presentation of tumour-associated antigens by HLA-DR has been hypothesised [32]. Scarlett and colleagues show that significant dysfunction in dendritic cells (including MHC class II loss) drives cancer development and metastasis in a mouse model of inducible human cervical cancer [39]. Interestingly, T cells in this model retained the ability to recognise and respond to antigenic stimuli, even at late cancer stages, whereas dendritic cells become dysfunctional upon cancer progression. These observations highlight the critical importance of effective antigen presentation for control of carcinoma progression, suggesting that T cell priming, a key step in the cancer-immune set point [40], may be just as important as traditional immune checkpoints [41]. Indeed, HLA-DR may itself represent an important and currently underutilised immunotherapeutic target.

### Electronic supplementary material

Below is the link to the electronic supplementary material.

**Supplementary Figure** **1**. HLA-DR is not significantly altered with lymphovascular invasion in a cohort of stage II CRC patients. No significant associations were found between percentage HLA-DR expression and presence of lymphovascular invasion (LVI), in carcinoma adjacent stroma (A) or epithelium (B), or in carcinoma stroma (C) or epithelium (D) in a cohort of n = 61 stage II CRC carcinomas. ns = non-significant. **Supplementary Figure** **2**. Correlating HLA-DR expression with a number of carcinoma buds in a cohort of stage II CRC patients. No significant associations were found between percentage HLA-DR expression and number of carcinoma buds, in carcinoma adjacent stroma (A) or epithelium (B), or in carcinoma stroma (C) or epithelium (D) in a cohort of n = 30 stage II CRC carcinomas with budding data. (PDF 100 kb)
