# Supplementary material for: Characterising the prognostic potential of HLA-DR during colorectal cancer development
Source: Cancer Immunol Immunother. 2020 Apr 18;69(8):1577–88. doi: 10.1007/s00262-020-02571-2 (PMC7347515; doi:10.1007/s00262-020-02571-2)

Supplemental Figure 1.

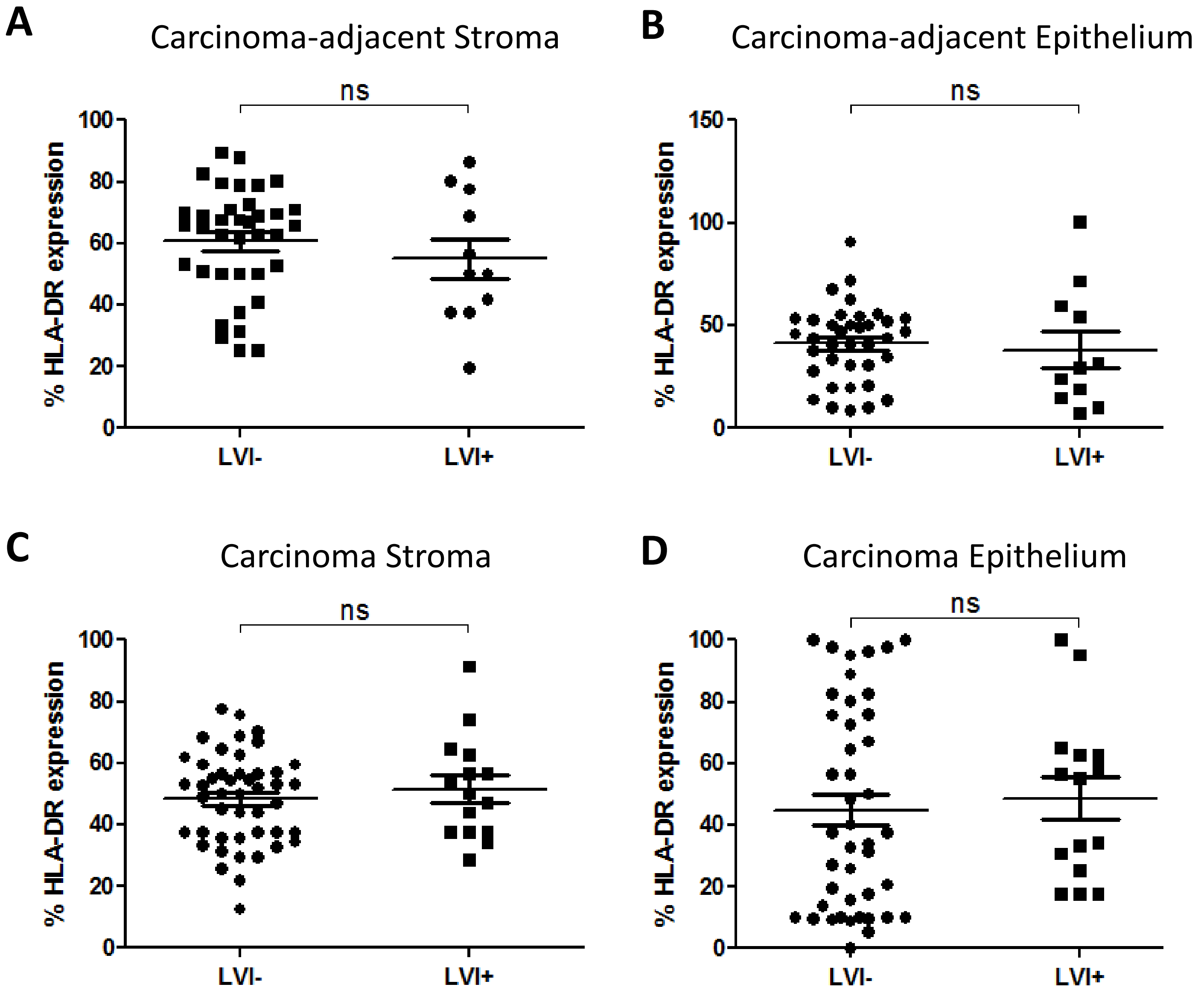

Supplemental Figure 2.

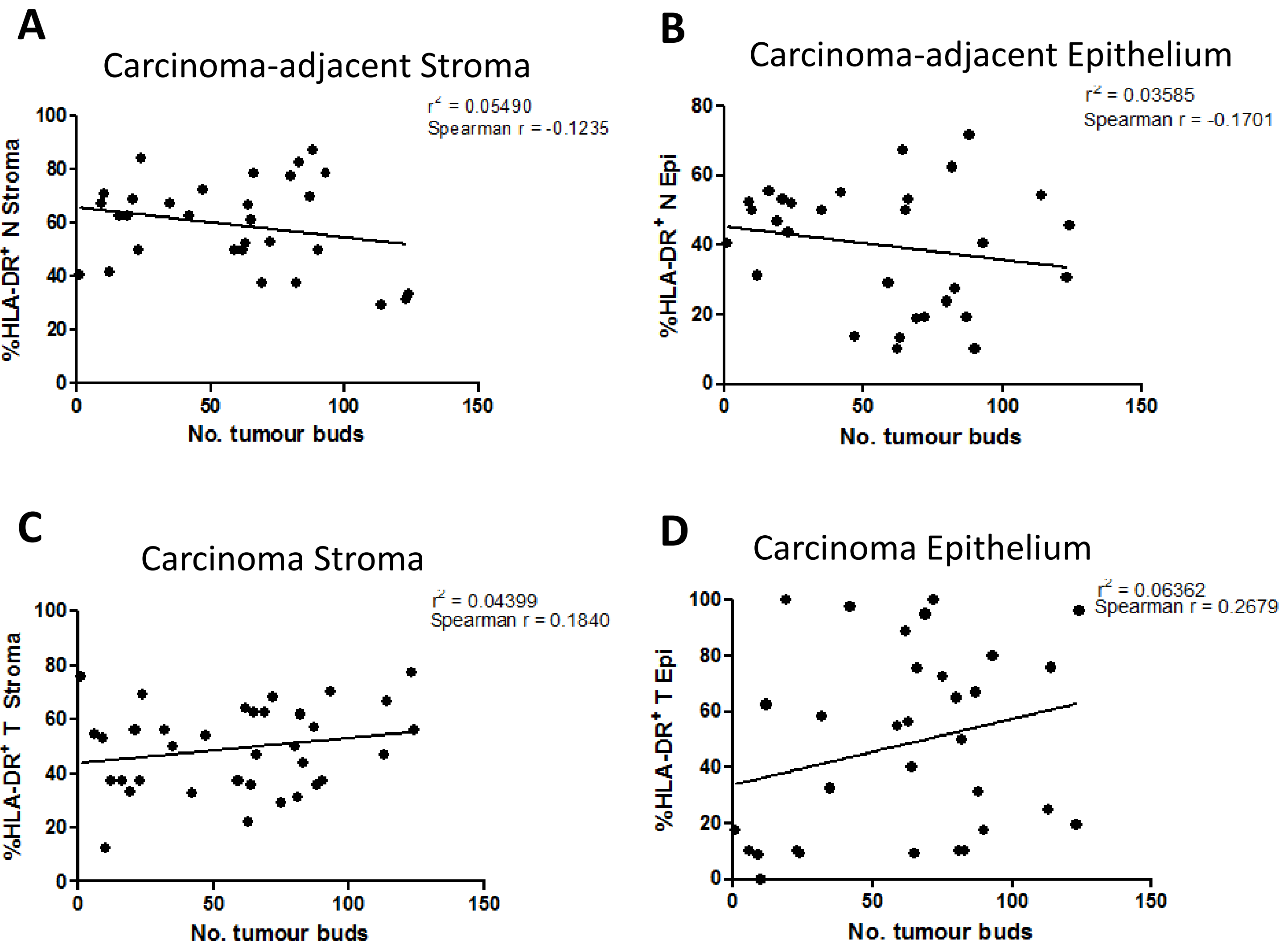

Supplement: Supplementary file 1 — Supplementary Figure 1. HLA-DR is not significantly altered with lymphovascular invasion in a cohort of stage II CRC patients. No significant associations were found between percentage HLA-DR expression and presence of lymphovascular invasion (LVI), in carcinoma adjacent stroma (A) or epithelium (B), or in carcinoma stroma (C) or epithelium (D) in a cohort of n = 61 stage II CRC carcinomas. ns = non-significant. Supplementary Figure 2. Correlating HLA-DR expression with a number of carcinoma buds in a cohort of stage II CRC patients. No significant associations were found between percentage HLA-DR expression and number of carcinoma buds, in carcinoma adjacent stroma (A) or epithelium (B), or in carcinoma stroma (C) or epithelium (D) in a cohort of n = 30 stage II CRC carcinomas with budding data. (PDF 100 kb) [file 262_2020_2571_MOESM1_ESM.pdf]
